# A Soothing Lavender-Scented Electrospun Fibrous Eye Mask

**DOI:** 10.3390/molecules29225461

**Published:** 2024-11-19

**Authors:** Dandan Kang, Yichong Li, Xiaowen Dai, Zixiong Li, Kai Cheng, Wenliang Song, Deng-Guang Yu

**Affiliations:** School of Materials and Chemistry, University of Shanghai for Science and Technology, Shanghai 200093, China; 232223076@st.usst.edu.cn (D.K.); 2035052114@st.usst.edu.cn (Y.L.); 2135070612@st.usst.edu.cn (X.D.); 2135070417@st.usst.edu.cn (Z.L.); 2135070412@st.usst.edu.cn (K.C.)

**Keywords:** electrospinning, eye mask, lavender oil

## Abstract

Electrospinning technology has demonstrated extensive applications in biomedical engineering, energy storage, and environmental remediation. However, its utilization in the cosmetic industry remains relatively underexplored. To address the challenges associated with skin damage caused by preservatives and thickeners used for extending the shelf life of conventional products, a soothing lavender-scented electrospun fibrous eye mask with coaxial layers was developed using the electrospinning technique. Polyvinyl alcohol (PVA) served as the hydrophilic outer sheath, while polycaprolactone (PCL) constituted the hydrophobic core, with lavender oil (LO) encapsulated within. The structural and physicochemical properties of the samples were characterized using a scanning electron microscope (SEM), Fourier transform infrared spectroscopy (FT-IR), and contact angle measurements. Upon hydration, the fibrous membrane exhibited strong adhesion properties, notable antioxidant activity, and a degree of antibacterial efficacy, demonstrating its potential for safe and effective use in skincare and eye mask applications. These findings suggest that the developed electrospun material offers promising functional properties and functional properties for integration into cosmetic formulations.

## 1. Introduction

In recent years, as living standards have improved, the demand for skincare and aesthetic products has surged, with sheet masks emerging as a major growth segment in the skincare industry [1]. Conventional sheet masks, typically made from non-woven or biofiber materials, are impregnated with a serum containing active ingredients [2]. However, to maintain internal moisture and extend shelf life, these products often require the addition of preservatives, emulsifiers, and thickeners, which can potentially cause skin irritation [3,4,5]. Consequently, preservative-free dry masks have gained popularity as an effective solution to address this issue. This trend is particularly evident in eye skincare products, which are highly sought after by female consumers and individuals who spend extended periods working in front of screens. Due to the heightened sensitivity of the under-eye skin, it is essential to use natural ingredients that do not cause irritation or damage [6]. Ideally, these eye masks should be composed of skin-friendly materials with combined antibacterial, antioxidant, and soothing properties to promote relaxation and skin health [7,8].

Developing dry eye masks offers a dual advantage: eliminating the need for potentially harmful preservatives and achieving controlled release of functional ingredients. When activated with water, these masks exhibit strong adhesion, making them convenient for both daily use and travel. Thus, identifying an efficient method for fabricating functional dry eye masks with these properties has become a significant focus in cosmetic research and development [9]. Electrospinning is an advanced fabrication technique that utilizes high-voltage electric fields to stretch and transform polymer droplets into a cone-like shape, known as the Taylor cone, producing microfibers and nanofibers through solidification and deposition [10,11,12]. This method enables the formation of nanofibrous membranes characterized by unique properties such as a high surface-area-to-volume ratio, high porosity, small pore sizes, and tunable mechanical strength [13,14]. Due to these distinctive features, electrospun membranes are suitable for loading with a variety of bioactive compounds [15], including plant extracts with anti-inflammatory and antioxidant properties, making them highly versatile for applications in wound dressings, food packaging, and air filtration [16,17]. Despite these advantages, the potential of electrospinning in the cosmetic field remains underutilized.

In cosmetics, electrospinning offers a novel approach by creating dry nanofiber membranes infused with active ingredients that remain stable until the point of use [18,19,20]. Upon contact with the skin, the nanofibers undergo hydration, triggering the immediate release of active molecules directly onto the targeted skin surface. This mechanism eliminates the need for preservatives while ensuring the active components retain their potency and efficacy until application [21]. An advanced variant of this technique, coaxial electrospinning, enables the encapsulation of multiple functional components within a single fiber, resulting in a multicomponent structure that significantly enhances the functional and structural properties of the membrane [22]. Coaxial electrospun membranes possess a unique core–sheath structure, where the outer hydrophilic sheath layer facilitates rapid swelling in water, allowing the active ingredients to adhere effectively to the skin [23,24,25]. Meanwhile, the hydrophobic inner core reduces water evaporation, thereby protecting the internal components and prolonging the duration of active ingredient release. This hierarchical architecture not only improves moisture retention and skin adherence but also optimizes the controlled delivery of functional molecules, making it highly beneficial for sensitive areas such as the under-eye region.

Polyvinyl alcohol (PVA), a water-soluble synthetic polymer, is widely used in electrospinning due to its excellent film-forming ability, biocompatibility, and non-toxicity [26]. As a fiber-forming polymer, PVA enhances the structural integrity, moisture retention, and flexibility of electrospun membranes, making it an ideal candidate for cosmetic applications. On the other hand, polycaprolactone (PCL), a biodegradable and biocompatible polyester, is favored for its prolonged degradation time, superior mechanical strength, and elasticity [27]. PCL-based nanofiber membranes are frequently utilized in tissue engineering, drug delivery, and wound healing. In cosmetic formulations, PCL’s diffusion mechanism and low hydrophilicity and slow degradation rate allow for sustained and controlled release of active ingredients, contributing to long-term skin benefits while maintaining a robust and flexible membrane structure [28,29]. The combined use of PVA and PCL in coaxial electrospinning creates a synergistic effect, enhancing both the immediate and sustained release of active compounds, thereby offering an innovative solution for advanced skincare treatments.

The incorporation of various plant extracts into nanofiber membranes can endow the fibers with diverse functional properties, making them suitable for targeted applications [12]. Among these, lavender oil (LO), derived from the flowers of *Lavandula angustifolia*, is a widely recognized essential oil used extensively in cosmetics, aromatherapy, and skincare due to its multifaceted therapeutic properties [30]. Renowned for its calming aroma that alleviates stress and anxiety, LO also exhibits significant skincare benefits, including anti-inflammatory, antimicrobial, and antioxidant activities [31,32]. Its application can help soothe skin irritation and redness, combat acne through its antibacterial properties, promote wound healing and tissue regeneration, and protect against oxidative stress, thereby reducing signs of aging and enhancing skin health [33].

Given that both PVA and PCL have been established as non-toxic and biocompatible materials and LO is a naturally derived bioactive agent, this combination demonstrates strong potential for use in beauty and care of the skin under the eyes. In this study, bilayer PVA/LO*PCL nanofibrous membranes were fabricated using coaxial electrospinning technology. The structural and morphological characteristics of the membranes were comprehensively analyzed to confirm the successful fabrication of coaxial nanofibers, while the antioxidant efficacy and antibacterials ability of the membranes were systematically evaluated. The results provide a promising reference for the utilization of coaxial nanofibrous membranes in the cosmetic industry, highlighting their potential as advanced delivery systems for natural active ingredients in skincare and especially for eye mask applications.

## 2. Results and Discussion

### 2.1. Strategy for the Preparation of the Eye Mask

**Figure 1:**
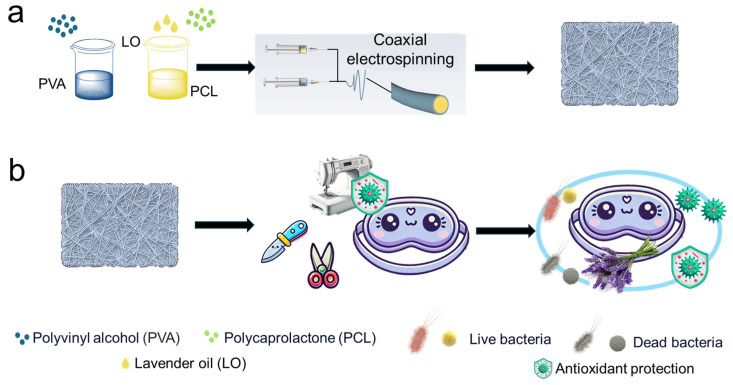
Coaxial electrospinning and core–shell nanofibers: (**a**) a diagram of the coaxial electrospinning system equipped with three primary components and (**b**) the design strategy for eye masks based on core–shell nanofibers membranes.

As shown in Figure 1, the eye mask samples were fabricated using coaxial electrospinning. First, PVA and LO-containing PCL solutions were prepared separately by dissolving PVA in distilled water and PCL in the appropriate solvent with different content of LO (0.1, 3.5, and 5.0 wt.%) under continuous stirring. For coaxial electrospinning, the PVA solution was used as the sheath layer, while the LO-containing PCL solution served as the core layer. The resulting coaxial fibers, designated as PVA@LO*PCL, were fabricated using a custom-built electrospinning setup. Meanwhile, uniaxial PCL, PVA, and PVA@PCL fiber membranes were also produced as control samples for comparison.

### 2.2. Characterization of the Fibrous Membrane of the Eye Mask

**Figure 2:**
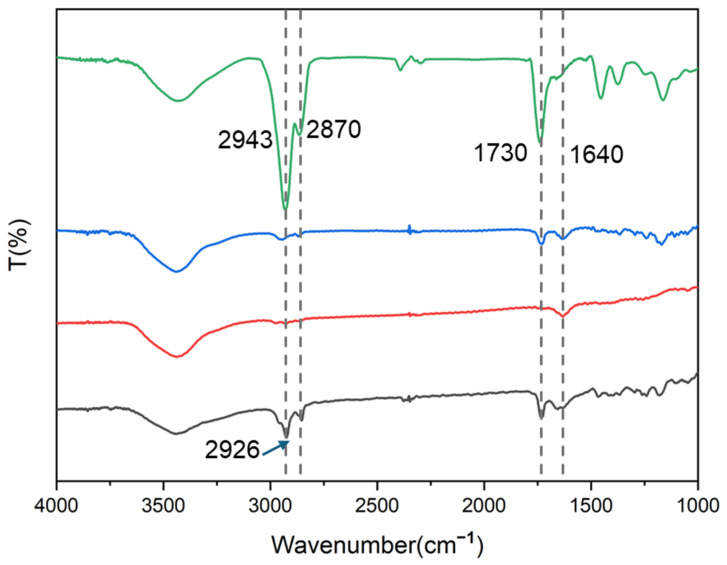
The FT-IR spectra of the nanofiber membrane.

To confirm the successful fabrication of the PVA@LO*PCL fibers, the Fourier transform infrared spectroscopy (FT-IR) spectra of PCL and PVA were analyzed. In the FT-IR spectrum of PCL, the absorption peak at 2926 cm^−1^ corresponded to the bending vibration of saturated C-H, while the peak at 2870 cm^−1^ was attributed to -CH_3_ [34]. Additionally, the absorption peak at 1730 cm^−1^ was the bending vibration peak of lactones. In the FT-IR spectrum of PVA, a prominent peak at 3200 cm^−1^ was assigned to the bending vibration of -OH, and the peak at 1200 cm^−1^ was linked to the C-O bending vibration [35]. In the LO spectrum, the absorption peak at 2943 cm^−1^ came from -CH_2_, and the absorption peak at 1730 cm^−1^ came from C=O. From Figure 2, it can be observed that in the spectrum of PVA@LO*PCL, the absorption peaked at 2926 cm^−1^ for -CH_2_- shifted to 2943 cm^−1^, originating from LO and PCL. The C=O peak at 1730 cm^−1^ from PCL and ester groups in LO, along with the C-O and -OH peaks from PVA, were also present in PVA@LO*PCL, but the absorption peaks were not obvious, possibly because LO was encapsulated in a coaxial structure.

**Figure 3:**
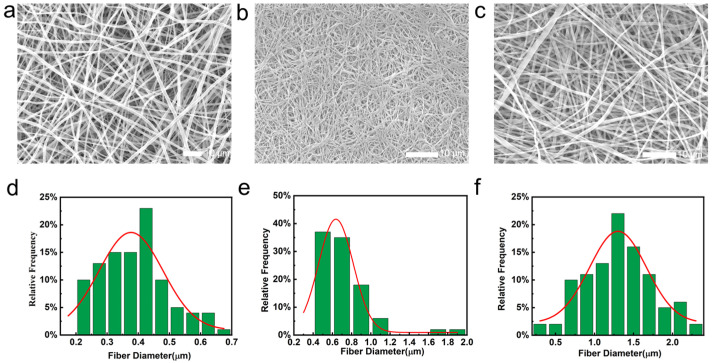
The fiber showing the SEM images and fiber diameter distributions of the fiber membranes ((**a**,**d**) PVA, (**b**,**e**) PCL, and (**c**,**f**) PVA@PCL).

Figure 3 illustrates the scanning electron microscope (SEM) images of the fabricated fibers. Figure 3a shows the SEM image of PVA nanofiber membranes, where the fibers were randomly distributed, intertwined, and formed a three-dimensional network structure with an average fiber diameter of around 400 nm. In the process of electrospinning, the instability of spinning voltage may lead to uneven fiber diameter and thus uneven fiber thickness. The high viscosity of the PVA solution will cause the fiber to stretch unevenly during the formation process, resulting in a rough surface. The inhomogeneity and roughness of the fibers will lead to the degradation of the quality of the film. Figure 3b displayed the SEM image of pure PCL fibers, which had a larger fiber diameter compared with PVA fibers. These PCL fibers had a smooth, uniform, and cylindrical surface, with no broken filaments or beads, indicating good film formation.

**Figure 4:**
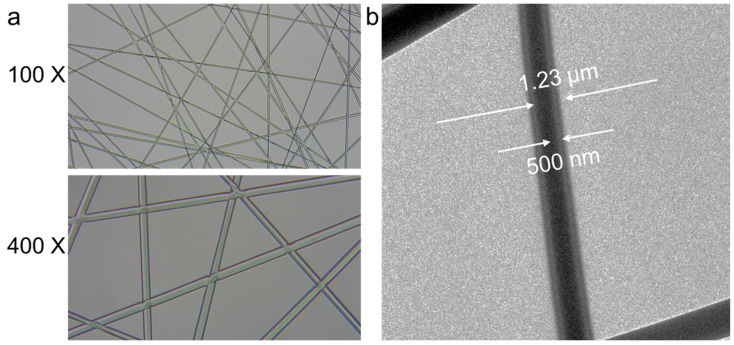
The fiber showing the (**a**) optical and (**b**) TEM images of PVA@LO*PCL by the coaxial electrospinning methods.

Figure 3c shows the SEM image of PVA@LO*PCL coaxial nanofiber membranes. Due to the coaxial electrospinning, the fiber diameter was larger than that of PVA or PCL fibers alone. The coaxial nanofibrous membrane exhibited a uniform fiber diameter and well-defined morphology, with an average diameter of 1.3 µm. To further verify the successful fabrication of coaxial fibers, optical and transmission electron microscopy (TEM) images were obtained (Figure 4). The optical microscope images showed a distinct color contrast between the inner and outer layers of the fibers. TEM images further confirmed the bilayered coaxial structure, revealing an inner fiber diameter of approximately 500 nm and an outer diameter of about 1.23 µm. These observations strongly supported the successful formation of coaxial PVA@LO*PCL nanofibers.

**Figure 5:**
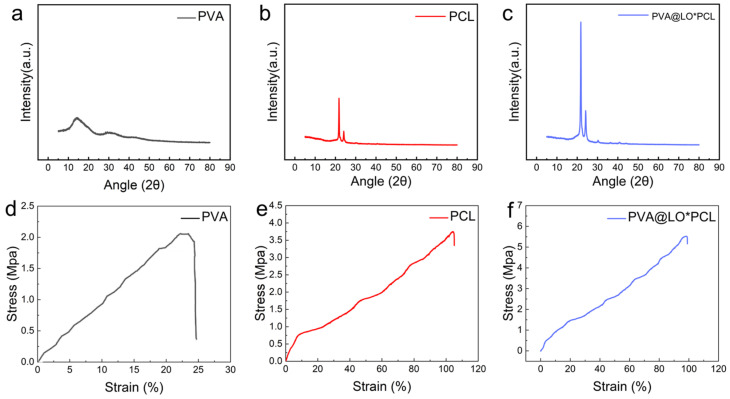
XRD and stress–strain curves of (**a**,**d**) PVA, (**b**,**e**) PCL, and (**c**,**f**) PVA@PCL.

The X-ray diffraction (XRD) patterns of PVA, PCL, and PVA@PCL nanofibrous membranes are presented in Figure 5a–c. The XRD profile of PVA displayed no discernible peaks, indicating its amorphous nature (corresponding to the ICDD PDF card number 00-033-1535). In contrast, PCL exhibited a prominent sharp peak at 21.3° and a secondary, lower-intensity peak at 23.6° (2θ). Consistent with the ICDD PDF card number 00-060-0295, it corresponded to the (110) and (200) crystal planes of PCL, further verifying the semi-crystalline structure of PCL. The XRD pattern of PVA@PCL demonstrated the presence of all characteristic peaks corresponding to PCL, confirming the retention of the crystalline nature within the composite scaffold. However, a noticeable reduction in the intensity of PCL peaks was observed, suggesting a decrease in crystallinity for PVA@PCL. This reduction was likely attributed to the interaction between PCL and PVA, which disrupted the crystalline packing of PCL in the composite matrix. As shown in Table 1, the tensile stress of PCL was 4.33 ± 1.45 MPa, which was much higher than that of PVA (1.55 ± 0.78 MPa), and PCL also had a higher elastic modulus (10.82 ± 1.65 MPa) and elongation at break (51.24 ± 3.57%). This was attributed to the higher molecular weight of PCL, whereas PVA, being a hydrophilic polymer, easily absorbed water, leading to a decrease in its mechanical properties. Figure 5d–f show the mechanical properties of the nanofibrous membranes, as evidenced by their ultimate tensile strengths. The PVA nanofibrous membrane exhibited an ultimate tensile strength of 2.21 MPa, whereas the PCL nanofibrous membrane showed a higher strength of 3.63 MPa. The PVA@PCL coaxial nanofibrous membrane displayed a significantly enhanced ultimate tensile strength of 5.72 MPa. This improvement could be attributed to the uniform fiber morphology and the optimized diameter distribution achieved through coaxial electrospinning. The formation of a well-aligned coaxial structure with coarser fiber diameters resulted in improved load distribution, thereby enhancing the mechanical properties of the PVA@PCL core–shell nanofibrous membrane.

**Figure 6:**
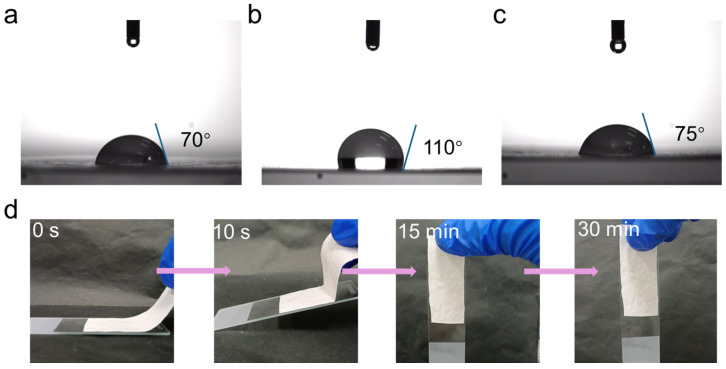
Contact angle images of (**a**) PVA, (**b**) PCL, and (**c**) PVA@PCL nanofiber membrane. (**d**) The adhesion of PVA@LO*PCL nanofiber membrane.

PCL is recognized for its hydrophobic nature, whereas PVA exhibits hydrophilic characteristics. As shown in Figure 6a–c, the contact angle of PVA was measured at 70°, indicating moderate hydrophilicity, while PCL demonstrated a significantly higher contact angle of 110°, confirming its strong hydrophobicity. In the coaxial spun membrane, PVA was used as the sheath layer, contributing to an increase in the overall hydrophilicity of the film [36,37]. However, due to the presence of PCL in the core layer, the composite film exhibited intermediate hydrophobic properties compared with pure PVA, demonstrating the interplay between the two polymers [38]. The hydrophilicity of the nanofibrous membrane is critical for its performance in applications such as skin protection, moisture retention, and skin adhesion [39]. A membrane with suitable hydrophilicity can create a moist microenvironment around the eyes, which is advantageous for eye hydration and facilitates controlled release of the internal LO. The hydrophobic nature of PCL in the inner core can effectively encapsulate and protect the LO, ensuring sustained antimicrobial and antioxidant activity. Additionally, adhesion tests were conducted on fiber membranes formed by sequential electrospinning, using the PVA layer as the outer sheath. When the PVA layer made contact with water, the fibers swelled and rapidly transformed into a transparent gel, disrupting the fiber structure and exposing the PCL layer to the liquid medium. This facilitated the controlled release of the encapsulated LO. The modified sheath structure demonstrated excellent adhesive properties. When the nanofibrous membrane was placed on a moistened clean slide, the PVA sheath layer rapidly adhered to the surface by forming a gel-like interface between the slide and the inner PCL hydrophobic layer. Figure 6d illustrates that after 15 min, even as the water began to evaporate, the nanofibrous membrane maintained good adhesion to the slide. After 30 min, once the water evaporated completely, the film remained firmly attached, highlighting the stability and strong adhesion properties of the membrane. This behavior demonstrated the potential of the nanofibrous membranes for use in eye-surrounding skin dressings, providing effective hydration, prolonged retention, and controlled release of active ingredients.

The primary antimicrobial activity of the PVA@PCL coaxial nanofiber membrane was attributed to the incorporation of LO. Lavender, a sub-shrub belonging to the Lamiaceae family, is primarily cultivated in the Ili region of China. The main antimicrobial compound in lavender essential oil is linalool, which disrupts bacterial cell membranes, effectively inhibiting bacterial growth and reproduction [40,41,42]. In addition to linalool, LO contains active volatile components such as camphor and linalyl acetate, which also exhibit significant antimicrobial properties and can prevent and treat a variety of bacterial infections [33,43]. Therefore, antibacterial and antioxidation experiments were conducted to evaluate the potential efficacy of the PVA@LO*PCL nanofiber membrane as an eye mask material.

### 2.3. Antimicrobial and Antioxidant Study

**Figure 7:**
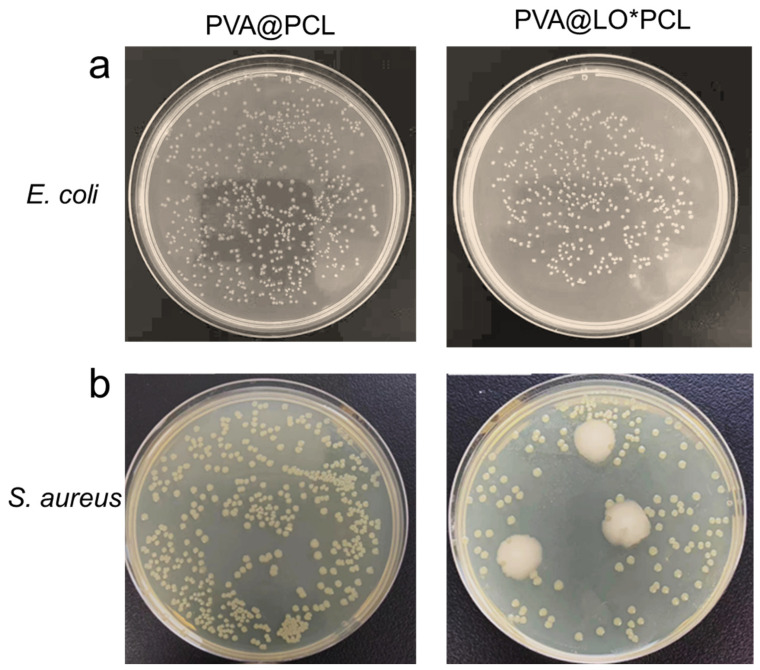
Photographs of bacterial culture plates of (**a**) *E. coli* and (**b**) *S. aureus* following treatment with PVA@PCL and PVA@LO*PCL fibrous membranes.

Figure 7a demonstrated that the PVA@PCL fiber membrane, lacking LO, exhibited no antibacterial activity against *Staphylococcus aureus* (*S. aureus*) and *Escherichia coli* (*E. coli*), showing results comparable to a blank plate. In contrast, Figure 7b shows that the PVA@LO*PCL nanofiber membranes significantly reduced the number of bacterial colonies when co-cultured with these bacteria, indicating a noticeable antimicrobial effect due to the presence of LO [44]. Although some bacterial colonies were still observed, their numbers were substantially lower compared with the PVA@PCL membranes, confirming the partial antibacterial capacity of LO.

**Figure 8:**
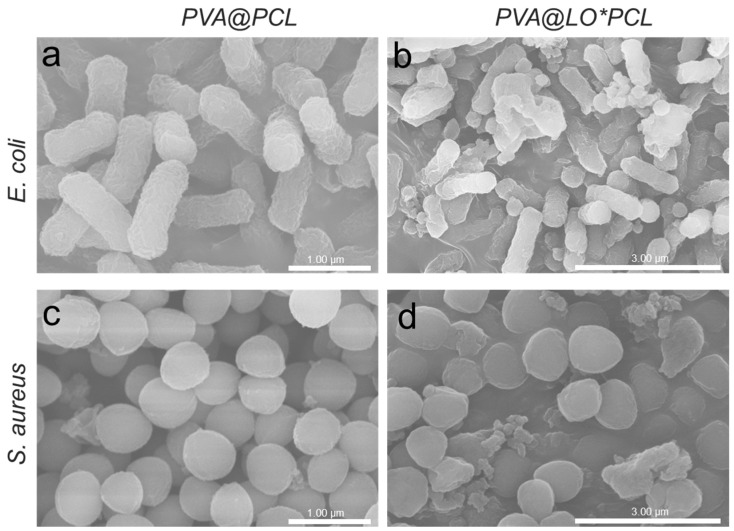
Photographs of bacterial culture plates of (**a**,**b**) *E. coli* and (**c**,**d**) *S. aureus* following treatment with PVA@PCL and PVA@LO*PCL fibrous membranes.

To further elucidate the antimicrobial mechanism of the PVA@LO*PCL fibers, SEM analysis was performed on the bacteria co-cultured with the nanofiber membranes (Figure 8). The SEM images revealed a large number of bacterial cells with disrupted and deformed cell membranes on the PVA@LO*PCL membrane, indicating severe morphological damage and bacterial death. In contrast, the bacterial cells on the PVA@PCL membrane without LO retained intact cell morphology and remained viable. This phenomenon was consistently observed in both *E. coli* and *S. aureus*, suggesting the broad-spectrum antimicrobial efficacy of the PVA@LO*PCL fibers. The observed antibacterial effect is likely due to the controlled release of LO from the coaxial nanofibers. LO, once released from the core layer, disrupted the bacterial cell membrane structure, leading to cell lysis and death. This study confirmed that the PVA@LO*PCL coaxial nanofiber membrane possessed significant antimicrobial properties, making it a promising candidate for applications in eye-surrounding skin dressings and related antibacterial products.

**Figure 9:**
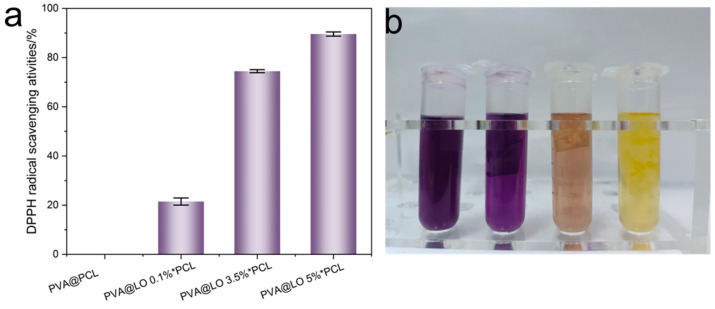
(**a**) DPPH radical scavenging rate and (**b**) solution change color for different concentrations of PVA@LO*PCL nanofiber membranes.

The antioxidant activities of LO-loaded and non-LO-loaded nanofiber membranes were evaluated using the 1,1-diphenyl-2-picrylhydrazyl (DPPH) radical scavenging assay. As shown in Figure 9a, the PVA and PCL nanofiber membranes, without LO, exhibited no scavenging effect on DPPH radicals. In previous studies, LO was confirmed to possess strong antioxidant properties, with its scavenging efficiency against DPPH radicals increasing with higher concentrations. In the current study, the DPPH radical scavenging efficiency of the PVA@LO*PCL nanofiber membrane was found to be 21.46% ± 1.46% at a 1% LO concentration. When the concentration was increased to 5%, a noticeable change in the color of the DPPH solution to light yellow was observed, and the scavenging rate significantly increased to 89.57% ± 0.85% (Figure 9b), indicating the efficient release and activity of LO within the nanofiber matrix.

This finding suggests that PVA@LO*PCL composite nanofiber membranes can effectively release LO, exhibiting substantial antioxidant activity. The versatility of the composite nanofibers to function in varied conditions highlights their potential application as antioxidant-active eye masks, skin dressings, or encapsulating materials in cosmetics, biomedicine, or food preservation, where the controlled release of active components is essential for enhancing product efficacy and stability.

Lavender essential oil is well known for its soothing, antibacterial, and anti-inflammatory properties, making it a popular choice for treating acne-prone skin and irritated scalp conditions [40]. Its rich composition, which includes ketones and monoterpene alcohols, gives it unique purifying capabilities and the ability to regulate sebum production, a critical factor for maintaining healthy and balanced skin [42]. Scientific studies have demonstrated lavender essential oil’s effectiveness in reducing disease-causing bacteria, highlighting its potential as a natural alternative treatment for a variety of skin conditions. By alleviating inflammation and suppressing bacterial growth, LO contributes to clearer, blemish-free skin. Regular use of high-quality lavender essential oil, such as Super Lavender, can significantly improve skin texture and elasticity while offering natural protection against external pathogens. This positions LO as a cornerstone ingredient in sensitive and acne-prone skincare formulations, offering a gentle and natural alternative to harsh chemical treatments.

The encapsulation of therapeutic molecules in the core of electrospun fibers provides protection and controlled release, enhancing their stability and efficacy over time [45]. Electrospinning enables the incorporation of functional agents within the fiber matrix, allowing for a gradual and targeted release of the therapeutic agents to the affected area [46]. Successful drug encapsulation within electrospun fibers depends on the homogenous distribution of the active compounds throughout the fiber structure [15,47]. Factors such as drug stability, solubility, and fiber morphology play crucial roles in determining the efficiency of drug encapsulation and the subsequent release profile. To address these challenges, various types of electrospun fibers have been developed with diverse structures to optimize drug delivery systems [48,49,50]. These fibers protect the active molecules from environmental degradation and allow for precise modulation of release rates. Depending on the application, functional molecules can be either physically adsorbed onto the fiber surface (surface immobilization) or directly incorporated into the polymer solution before electrospinning [51,52].

The efficacy of an eye mask, especially for sleep and relaxation, lies in its ability to promote improved sleep quality and reduce stress by blocking external light and creating a soothing environment. In the present study, electrospun eye masks were fabricated using a classical coaxial electrospinning technique, followed by precise cutting into the shape of an eye mask. For practical use, these electrospun eye masks were soaked in lukewarm water for a few minutes to allow for swelling and activation of the lavender essential oil. Upon application, these eye masks provided a gentle, soothing effect around the eyes, with a controlled release of LO, enhancing the relaxation experience and offering therapeutic benefits. The gradual release of the encapsulated essential oil helped in soothing the eye area, providing moisture, and potentially reducing dark circles and puffiness, making them a promising alternative for both skincare and relaxation applications [53].

## 3. Materials and Methods

### 3.1. Materials

The materials and equipment used in the experiments included Poly(ε-caprolactone) (PCL, Mw = 80,000, Shanghai Macklin Biochemical Co., Ltd., Shanghai, China), Poly(vinyl alcohol) (PVA, Mw = 27,045, Shanghai Macklin Biochemical Co., Ltd., China), lavender oil, and 2,2,2-Trifluoroethanol (Shanghai Macklin Biochemical Co., Ltd., China). The utilized instruments were as follows: an electronic analytical balance (BSA224S-CW, Sartorius Stedim Biotech, Göttingen, Germany), a magnetic stirrer (MS-M-S10, DLAB Scientific Co., Ltd., Beijing, China), a micro syringe pump (KDS100, Cole-Parmer, Shanghai, China), a field emission scanning electron microscope (SEM, Quanta FEG, FEI Company, Hillsboro OR, USA), a Fourier transform infrared spectrometer (FT-IR, SPECTRUM100, Perkin Elmer, Shelton, CT, USA), and a contact angle meter (DSA30, KRüSS GmbH, Hamburg, Germany).

### 3.2. Preparation of Spinning Solutions and Fabrication of Nanofiber Membranes

The preparation of spinning solutions began with accurately weighing a measured amount of PCL into a reagent beaker, followed by the addition of 2,2,2-trifluoroethanol (TFE) as the solvent. The mixture was then stirred on a magnetic stirrer at 500 rpm until complete dissolution was achieved at room temperature, resulting in a homogeneous PCL solution. In a parallel process, a predetermined amount of PVA was weighed and mixed with deionized distilled water in another reagent bottle. The PVA solution was heated and stirred at 500 rpm on a magnetic stirrer at 80 °C until fully dissolved, yielding a clear and consistent PVA solution.

Prior to the fabrication of coaxial fibers, uniaxial electrospinning trials were conducted separately for each polymer to establish baseline nanofiber membranes of PCL and PVA. The electrospinning setup comprised a pressure supply unit, a liquid feed unit, and a collection unit. Each polymer solution was loaded into a syringe and mounted on a microsyringe pump. A positive voltage was applied to the needle tip, while the collection plate, covered with aluminum foil, was connected to a negative voltage. The electrospinning parameters, including flow rate and voltage, were fine-tuned to stabilize the spinning jet. Optimal flow rates were determined to be 0.2 mL/h for PVA and 1.2 mL/h for PCL, with respective applied voltages that ensured the formation of a stable Taylor cone.

The formation of uniform fibers was visually confirmed by the presence of a stable jet and consistent fiber deposition on the collector plate. After an appropriate duration of electrospinning, the nanofibrous membranes were collected, dried, and stored for further analysis. These uniaxial PVA and PCL nanofiber membranes served as reference controls for evaluating the morphology, mechanical properties, and structural characteristics in comparison with coaxially spun fibers produced in subsequent experiments.

### 3.3. Preparations of LO-Loaded Coaxial Nanofiber Membranes

PVA@PCL coaxial nanofibers were fabricated using a coaxial electrospinning technique. The experimental setup consisted of a pressure supply unit, a liquid delivery system, and a collection unit. Two syringe needles were employed for simultaneous extrusion of the core and sheath solutions, with 7 mL of PCL solution and 3.5 mL of PVA solution loaded into separate syringes and mounted onto a microsyringe pump. The solutions were delivered through a coaxial nozzle system, where the PVA served as the sheath solution and PCL as the core. Positive voltage was applied to both needles using clamps, while the collection plate, covered with aluminum foil, was connected to a negative voltage source to establish the electrospinning field. The flow rates for the PCL core and PVA sheath solutions were precisely maintained at a 2:1 ratio (2 mL/h for PCL and 1 mL/h for PVA), regulated through the system’s control panel. This ratio was critical for ensuring smooth coaxial jet formation and preventing bead formation. Stability of the spinning jet and formation of a uniform Taylor cone were achieved by optimizing the applied voltage. After a specified spinning duration, the as-spun coaxial PVA@PCL nanofibrous membranes were successfully collected on the aluminum-covered collector plate.

To fabricate the LO-loaded PVA@PCL membranes, a predetermined amount of LO was incorporated into the PCL core solution. The mixture was stirred thoroughly to achieve a homogeneous distribution of the oil within the PCL matrix. This LO-enriched PCL solution was then used as the core fluid in the coaxial electrospinning process, following the same protocol and conditions as for the pristine PVA@PCL fibers. The resulting LO-loaded PVA@PCL nanofibrous membranes exhibited a uniform morphology and consistent fiber structure, indicating successful encapsulation of LO within the PCL core. These LO-loaded membranes were intended for subsequent characterization and performance evaluation.

### 3.4. Characterization

An attenuated total reflection-Fourier transform infrared spectroscopy (ATR-FTIR) spectrophotometer (Thermo Fisher Scientific Co., Ltd., Stoughton, MA, USA) was used to examine the chemical structure of the fibrous membranes. The membranes were scanned directly in the range of 4000–500 cm^−1^ with a resolution of 4 cm^−1^. The fibrous membranes were cut into a rectangular shape of 5 mm × 1 cm and loaded onto a glass slide, and the contact angle of the membrane was tested using a DSA30 contact angle instrument (KRÜSS GmbH, Germany) equipped with a video capture system. The microscopic morphologies of the membranes were analyzed using a Quanta FEG (FEI, USA) scanning electron microscope (SEM). For SEM imaging, the fibrous membranes were mounted on aluminum stubs with double-sided adhesive carbon tape and coated with a thin layer of gold via sputtering to enhance conductivity. Transmission electron microscopy (TEM) images were recorded by placing the electrospun coaxial fibers onto carbon-coated copper TEM grids. Water contact angles of these membranes were measured using the DSA30 contact angle meter. The mechanical properties, specifically the tensile strength, of the fabricated membranes were evaluated using a universal testing machine.

The effect of nanofiber membranes on DPPH radical scavenging was evaluated using the DPPH radical scavenging assay. The antioxidant activity of PVA@LO*PCL nanofiber membranes was assessed by immersing 5 mg of PVA@LO*PCL membranes containing varying concentrations of LO into 3 mL of 0.2 mmol/L DPPH ethanol solution. The mixtures were stirred evenly and allowed to react for 30 min in the dark. After the reaction, the UV absorbance of the sample mixtures was measured at 517 nm using a UV-Vis spectrophotometer. The DPPH radical scavenging activity was calculated based on the decrease in absorbance, indicating the antioxidant capacity of the membranes.

$$\mathrm{DPPH}\;\mathrm{scavenging}\;\mathrm{activity}=\frac{A_{0}-A_{i}}{A_{0}}\times 100\%$$

where *A*_0_ is the absorbance of the untreated DPPH solution, and *A_i_* is the absorbance of the DPPH solution treated with the film.

### 3.5. Antimicrobial Activity

The antimicrobial activity of the nanofiber membranes was evaluated using the plate counting method. Two representative bacteria, *E. coli* (Gram-negative) and *S. aureus* (Gram-positive), were used in the experiments. The bacterial strains were initially cultured overnight in LB medium at 37 °C with constant shaking at 200 rpm, and the cell density was adjusted to 1 × 10^6^ CFU/mL. Film samples (2 cm × 2 cm) were incubated with the bacterial suspensions at 37 °C for 12 h. After incubation, 100 μL of each mixture was plated onto agar plates and incubated at 37 °C for an additional 12 h. The number of bacterial colonies formed on the agar plates was counted to determine the antibacterial efficacy of the membranes. All experiments were performed in triplicate.

To further investigate the effect on bacterial cell integrity, the treated and controlled bacterial cells were analyzed using SEM imaging. For treatment, bacterial strains were incubated with film samples at 37 °C for 12 h, while a parallel set of untreated strains served as the control. Following incubation, the bacterial cells were collected by centrifugation, rinsed with PBS, and fixed overnight at 4 °C in a 2.5% glutaraldehyde-paraformaldehyde solution. The fixed samples were then subjected to a graded ethanol dehydration process, with each concentration applied for 15 min. After dehydration, the samples were freeze-dried, and the surface morphology of the bacterial cells was visualized using SEM.

## 4. Conclusions

In this study, the PVA@LO*PCL coaxial nanofibrous membranes were successfully fabricated using a custom-built electrospinning setup with a specially designed spinneret, and their application in eye mask formulations was explored.

Further experimental results demonstrated that the incorporation of LO within the PVA@PCL coaxial nanofibrous membrane significantly enhanced its antimicrobial and antioxidant properties, compared with the non-LO-loaded membranes. This suggests that the functional nanofibrous membrane could serve as an effective delivery system for bioactive compounds in skincare and therapeutic applications.

Compared with conventional hydrogel-based or wet sheet masks, the electrospun fiber membranes offer rapid release of active ingredients, eliminating the need for pre-servatives and other additives that might trigger skin sensitivities. This makes electrospun membranes particularly suitable for cosmetic and biomedical applications where safety and biocompatibility are paramount. Despite the promising results, research on the use of electrospinning technology for loading active substances into cosmetic products is still in its infancy. Further investigations are warranted to explore the potential of electrospun nanofibrous membranes in skincare, particularly in the targeted delivery of natural, non-irritating active compounds for benefits such as whitening, anti-aging, and skin rejuvenation. The development of rapid-release electrospun masks could pave the way for next-generation skincare products, offering a novel solution that combines high efficacy, enhanced stability, and minimal irritation. Such innovations could lead to the creation of “rapid-release” masks tailored to deliver a variety of therapeutic and cosmetic benefits, providing a transformative approach in the field of advanced skincare and dermatological treatments.

## Figures and Tables

**Table 1:** Mechanical properties of PVA, PCL, and PVA@LO*PCL nanofiber membranes.

Samples	Tensile Stress (MPa)	Elastic Modulus (MPa)	Elongation at Break (%)
PVA	1.55 ± 0.78	4.52 ± 1.16	31.57 ± 4.33
PCL	4.33 ± 1.45	10.82 ± 1.65	51.24 ± 3.57
PVA@LO*PCL	4.09 ± 1.27	9.70 ± 1.33	47.71 ± 4.26

## Data Availability

Data that support the findings of this study are available on request to the authors.
